# Expanding the Medfly Virome: Viral Diversity, Prevalence, and sRNA Profiling in Mass-Reared and Field-Derived Medflies

**DOI:** 10.3390/v14030623

**Published:** 2022-03-17

**Authors:** Luis Hernández-Pelegrín, Ángel Llopis-Giménez, Cristina Maria Crava, Félix Ortego, Pedro Hernández-Crespo, Vera I. D. Ros, Salvador Herrero

**Affiliations:** 1 Department of Genetics and University Institute of Biotechnology and Biomedicine (BIOTECMED), Universitat de València, 46100 Valencia, Spain; luis.hernandez-pelegrin@uv.es (L.H.-P.); angel.llopis@uv.es (Á.L.-G.); m.cristina.crava@uv.es (C.M.C.); 2 Laboratory of Virology, Wageningen University and Research, Droevendaalsesteeg 1, 6708 PB Wageningen, The Netherlands; vera.ros@wur.nl; 3 Department of Microbial and Plant Biotechnology, Centro de Investigaciones Biológicas Margarita Salas, CSIC, 28040 Madrid, Spain; ortego@cib.csic.es (F.O.); pedro@cib.csic.es (P.H.-C.)

**Keywords:** Tephritidae, small RNA, narnavirus, nodavirus, reovirus, totivirus

## Abstract

The Mediterranean fruit fly (medfly), *Ceratitis capitata*, is an agricultural pest of a wide range of fruits. The advent of high-throughput sequencing has boosted the discovery of RNA viruses infecting insects. In this article, we aim to characterize the RNA virome and viral sRNA profile of medfly. By means of transcriptome mining, we expanded the medfly RNA virome to 13 viruses, including two novel positive ssRNA viruses and the first two novel dsRNA viruses reported for medfly. Our analysis across multiple laboratory-reared and field-collected medfly samples showed the presence of a core RNA virome comprised of Ceratitis capitata iflavirus 2 and Ceratitis capitata negev-like virus 1. Furthermore, field-collected flies showed a higher viral diversity in comparison to the laboratory-reared flies. Based on the small RNA sequencing, we detected small interfering RNAs mapping to all the viruses present in each sample, except for Ceratitis capitata nora virus. Although the identified RNA viruses do not cause obvious symptoms in medflies, the outcome of their interaction may still influence the medfly’s fitness and ecology, becoming either a risk or an opportunity for mass-rearing and SIT applications.

## 1. Introduction

Insect specific viruses (ISVs) specifically infect insect hosts. Some ISVs are highly pathogenic (e.g., baculoviruses infecting caterpillars [1]), while others cause low-level infections that have seemingly no effect on host fitness [2]. Recently, the wide application of high throughput sequencing has provided a continuous flow of newly discovered ISVs, specifically RNA viruses [3,4,5]. Those ISVs belong to diverse families of single and double strand RNA viruses, including *Iflaviridae*, *Nodaviridae*, *Negeviridae*, *Togaviridae*, *Rhabdoviridae*, *Reoviridae*, or *Totiviridae* [2,6,7,8].

Covert RNA viruses represent a subset of ISVs that are found to co-exist in an equilibrium with their host, in which virus vertical transmission is assured while no major detrimental fitness costs are caused to the host [9]. However, covert infections may still produce behavioral and physiological changes in the host, and those changes might be context-dependent [2,10]. In the case of the Mediterranean fruit fly (medfly), *Ceratitis capitata*, fitness studies have shown that Ceratitis capitata nora virus (CcaNV) infection is correlated with a reduced adult lifespan [10]. These examples highlight the vast diversity of the effects caused by RNA viral infections and indicate the potential application of RNA viruses as biological control agents against insect pests.

*Ceratitis capitata* (Diptera: Tephritidae) is a polyphagous species causing economic losses in agriculture worldwide. The larvae of this species can feed on a wide range of fruits including citrus fruits (*Citrus* spp.), apples (*Malus pumila*), or pears (*Pyrus communis*) (EPPO Global Database; https://gd.eppo.int, accessed on 6 October 2021). Sterile insect technique (SIT) programs have been applied for medfly control in North America, Central America, South America, Europe, the Middle East, Asia, Africa, and Australia [11]. This strategy relies on the systematic area-wide release of sterile males obtained from mass-rearing facilities. Mass-rearing facilities offer a controlled way to rear large quantities of insects for different purposes. However, such mass-reared insects are threatened by viral diseases, coming from viruses introduced via horizontal transmission or from already present covert infections that are triggered into overt infections [12,13].

In recent years, up to nine RNA viruses have been identified in *C. capitata* and all of them seem to be present in a covert state, not causing clear symptoms [7,8,10,14]. One of these viruses has a negative single-stranded (ss) RNA genome, Ceratitis capitata sigmavirus (CcaSV), which belongs to the *Rhabdoviridae* family [14]. The other eight viruses have a positive ssRNA genome and can be divided into two groups. Three viruses relate to the insect-infecting virga-negev-like virus group, Ceratitis capitata transcript shotgun assembly 1 (CcaTSA1), Ceratitis capitata transcript shotgun assembly 2 (CcaTSA2), and Ceratitis capitata transcript shotgun assembly 3 (CcaTSA3) [8]. The second group is composed of five viral sequences phylogenetically classified within the *Picornavirales* order: Ceratitis capitata iflavirus 1 (CcaIV1), Ceratitis capitata iflavirus 2 (CcaIV2), Ceratitis capitata iflavirus 3 (CcaIV3), Ceratitis capitata iflavirus 4 (CcaIV4), and CcaNV [7,10]. Up until now, no double-stranded (ds) RNA viruses had been discovered in *C. capitata*.

In contrast to vertebrates, no evidence of adaptative immunity in insects has yet been provided [15]. Instead, insects rely on innate immunity, which is normally activated in response to external challenges [16]. Major mechanisms of innate immunity include the Toll Dorsal pathway (Toll), Janus kinase/signal transducer and activator of transcription pathway (JAK/STAT), immune deficiency pathway (Imd), and RNA interference (RNAi) [17]. RNAi is an evolutionarily conserved defense mechanism based on the use of small RNAs (sRNAs) that guide a protein-effector complex to target specific RNA through sequence-complementarity. Three distinct classes of sRNAs are known: microRNAs (miRNAs), small-interfering RNAs (siRNAs), and PIWI-interacting RNAs (piRNAs) [18,19]. While the main function of the miRNA pathway is the post-transcriptional regulation of the host gene expression, the siRNA pathway actively restricts viral infection by the recognition and cleavage of dsRNA molecules produced during viral replication. The production of viral siRNAs (vsiRNAs) against covert RNA viruses has been observed in dipteran species like the mosquito *Aedes vexans arabiensis* and the oriental fruit fly *Bactrocera dorsalis* [6,20]. The piRNA pathway was initially described as a silencing mechanism of transposable elements (TEs) in the gonadal tissues of *D. melanogaster* [21]. The potential antiviral role of the piRNA pathway has been discussed in some studies describing the presence of viral-derived piRNAs (vpiRNAs) in mosquitoes, but direct proof is still lacking [17,22].

In this study, high throughput sequencing and data mining were used to characterize the RNA virome of *C. capitata*, identifying new viral species. Further analysis of viral prevalence as well as viral small RNA profiling in reared and field-collected medfly samples additionally revealed a high diversity in viral abundance in different populations, and presented important insight into mechanisms of insect defense against RNA viruses in this pest.

## 2. Materials and Methods

### 2.1. C. capitata Samples and Cell Line

Two medfly laboratory strains (control and W-1Kλ) were used for transcriptomic analysis by RNA-seq and further virus discovery. Moreover, viral detection and abundance were determined in vivo in the pupae of two long-term laboratory-reared medfly strains (control and IVIA), one recently field-derived strain (Wild-F4), and one mass-reared strain (V8A). In total, five different medfly strains were used, and their origin and rearing are described below.

The control strain (C) originates from flies collected from untreated experimental fields at the “Instituto Valenciano de Investigaciones Agrarias” (IVIA, Moncada, Valencia, Spain) in 2001, and it has been reared for more than 100 generations under laboratory conditions (26 °C, 40 to 60% humidity, 14/10 h light/dark cycles) at the “Centro de Investigaciones Biológicas Margarita Salas” (CSIC, Madrid) [23].

The IVIA strain (I) originates from the control strain and is regularly renewed with field-captured flies from the experimental fields at the IVIA. The IVIA strain has been reared under the above-mentioned laboratory-rearing conditions for more than 30 generations.

Lambda-cyhalothrin resistant strain (W-1Kλ) started with flies collected from citrus orchards treated with malathion in Castellón (Valencia, Spain) in 2004, and first were selected for resistance to malathion for 39 generations and then for resistance to lambda-cyhalothrin for 43 generations after. The W-1Kλ strain is maintained under the above mentioned laboratory-rearing conditions at the facilities of CSIC (Madrid) [23].

The recently field-derived Wild-F4 strain (W) originates from field-collected *C. capitata* pupae obtained from infested figs fruits (*Ficus carica*) collected from isolated trees in commercial citrus orchards in Alcira (Valencia, Spain) in August 2020. The pupae used in this study were obtained from the F4 generation in December 2020. This colony was maintained daily at the facilities of the IVIA by TRAGSA (Empresa de Transformación Agraria, Valencia, Spain).

The Vienna 8A (V8A) strain is a naturalized V8-strain obtained by crossing the temperature sensitive lethal genetic sexing strain Vienna-8 mix 2002 with individuals collected in citrus orchards located in the province of Valencia (Spain). This medfly strain is currently mass-reared for application in SIT control in the province of Valencia, and was kindly provided by TRAGSA.

The Cce/cc128 cell line, which was developed in 1981 from *C. capitata* fertilized eggs [24], was kindly provided by José Galián (Department of Zoology and Physical Anthropology, University of Murcia) and was cultured in Shields and Sang culture medium (Sigma-Aldrich^®^, St Louis, MO, USA) with 10% FBS (PAA) and 100 U/mL streptomycin/penicillin at 25 °C.

### 2.2. Transcriptome Assembly and Virus Discovery

The presence of RNA viruses was analyzed in six transcriptome data sets obtained from the control and W-1Kλ strains (acc. numbers ERR4690327, ERR4690326, ERR4690325, SRR16562767, SRR16562768, and SRR16562769). The total RNA was extracted and used for cDNA synthesis and high-throughput sequencing by Illumina HiSeq 2000 paired-end sequencing, as described before [25]. For virus mining, first, the quality of the sequencing reads was analyzed using fastQC (https://www.bioinformatics.babraham.ac.uk/projects/fastqc/, accessed on 1 December 2020). The adaptors were removed, and the low-quality sequences were trimmed using Trimmomatic [26]. The sequencing reads corresponding to each medfly strain, including three replicates per strain, were de novo assembled using Trinity-v2.9.0 [27]. Default parameters were used for all programs.

Viral presence in the assembled contigs of each medfly strain was assessed using tBLASTn (protein query against translated nucleotide database) with a threshold value of 1 × 10^−5^. As a query, we used the protein viral sequences deposited in GenBank, classified within the *Riboviria* domain (RNA viruses), and having “invertebrates” as the host. After tBLASTn, the putative viral contigs resulting from the analysis were manually filtered to remove (a) the contigs less than 2000 bp in length, (b) the contigs found in the reference genome of *C. capitata* after BLASTn (e-value cut off of 1 × 10^−100^), and (c) the contigs without open reading frames (ORFs) after evaluation with ORFfinder (https://www.ncbi.nlm.nih.gov/orffinder/, accessed on 9 September 2021). Viral length was further confirmed by mapping all reads against the putative viral candidates using BWA [28], revealing a high coverage at the 5′ and 3′ ends of the viral genome.

### 2.3. Annotation of Viral Genomic Structures and Phylogenetic Classification

Viral candidates were annotated running BLASTx against the non-redundant protein sequence database (NCBI). The ORFs contained in the viral fragments were determined and their translated sequences were queried against the non-redundant protein database using BLASTp and the Conserved Domain Architecture Retrieval Tool (CDART) in GenBank. The phylogenetic analysis was performed at the amino acid level. The RNA dependent RNA polymerase (RdRp) conserved domain was selected to construct the phylogeny. When no conserved domains were defined, the protein sequence of the ORF containing the RdRp was selected. The closest viral relatives for the putative viruses under analysis were identified in the NCBI nr database using BLASTp. Other viruses recently described for medfly or similar dipteran species and belonging to the same viral families were added to the analysis (Table S3).

Protein sequences were aligned using T-coffee mode “psi-coffee”, which aligns distantly related proteins using the homology extension [29]. Fragments poorly aligned at the 5′ and 3′ ends of the sequences were manually trimmed. IQtree2 was run to provide the best model for the alignment. Finally, the phylogenetic trees were inferred by maximum likelihood using IQtree2 with the selected model, default settings, and 1000 ultrafast bootstrap [30], and were visualized using iTOL [31].

### 2.4. Viral Detection and Quantification

The presence and relative abundance of RNA viruses in laboratory-reared, mass-reared, and field-collected flies were determined using molecular methods. For this purpose, the total RNA was isolated from individual pupa using the TriPure isolation reagent (cat. no. 11667157001; Roche, Mannheim, Germany) according to the manufacturer’s protocol. Then, 1 µg of total RNA was treated with 1 µL of DNAse I (Invitrogen^TM^ DNAse I) to digest the DNA residues. The purified RNA was reverse transcribed into cDNA using oligo (dT) primers and random hexamers as specified in the Prime-Script RT Reagent Kit (Perfect Real Time from Takara Bio Inc., Otsu Shiga, Japan). Viral presence was detected through RT-qPCR (StepOnePlus Real-Time, Applied Biosystems, Foster City, CA, USA). All reactions were prepared in a total volume of 20 µL using 5× HOT FIREpol EvaGreen qPCR Mix Plus (ROX) from Solis BioDyne (Tartu, Estonia). Primer pairs for virus detection were designed using Primer 3 software [32] (Table S1). Unspecific binding to medfly genome sequences and self-priming were discarded using BLASTn, and primer efficiencies were estimated for the newly designed primer pairs (Table S1). The ribosomal L23a gene of the medfly (Genbank acc: XM004518966) was amplified through qPCR as an endogenous control of the RNA concentration using available primers [10]. Viral relative abundance was calculated by comparison of the viral Ct values and L23a Ct values, according to the following formula, E^−Ct (virus)^/E^−Ct (L23a)^, where E represents an amplification factor dependent on primer efficiency, which is 2 for 100% efficiency.

Medfly transcriptome data available in the NCBI database were investigated in silico for viral presence and relative abundance. SRA reads representing diverse medfly populations (Table S2) were selected and mapped against the genomic sequences of the 13 medfly RNA viruses using Bowtie 2 v 2.3.5.1 [33] and RSEM v 1.3.1 [34] with default parameters. The log relative abundance of each transcript is reported relative to the medfly endogenous L23a gene.

We also assessed viral presence in the Cce/cc128 cell line. About 1 × 10^6^ cells were pelleted through centrifugation to eliminate the supernatant and resuspended in 500 µL of TriPure reagent. RNA extraction and virus quantification were performed as described above.

Expression data derived from the different analysis were represented using the heatmap.2 function from the gplots v 3.1.1 package within the R software suite [35]. 

### 2.5. Viral Pre-Purification from Medfly Pupae and Virus Detection and Quantification

Fifty pupae of each strain (Control, IVIA, Vienna 8A, and Wild-F4) were suspended in 5 ml of TE buffer (Tris-HCl, pH 8–8.6, 1 mM EDT) with 0.06% SDS, and were homogenized using T 50 digital ULTRA-TURRAX^®^. Pupae homogenate was filtered using a gauze to eliminate cellular debris and was clarified with 1 volume of chloroform. The resulting samples were maintained at 4 °C for 10 min before 5 min centrifugation at 4 °C and 9000× *g*. The aqueous phase was recovered after centrifugation and was concentrated with 0.5 M of NaCl and 10% PEG 6000. The resulting mixture was incubated overnight at 4 °C with constant shaking. Final centrifugation at 4 °C and 10,000× *g* for 1 h contributed to the formation of a pellet containing the pre-purified viruses. The supernatant was removed, and the pellet was resuspended in 2 mL of PBS. Aliquots of 50 µL were used for RNA extraction and virus quantification as described above. For viral pre-purifications, the viral relative abundance was expressed in terms of viral genomes per µg of RNA. To obtain these rates, viral Ct values from the RT-qPCR were compared to the standard curves calculated for a fixed number of viral genome copies cloned in the pGEMTeasy vector [10].

### 2.6. Small RNA Sequencing

The control and Wild-F4 medfly strains were used for the small RNA analysis. Those strains were representatives of the highest (Wild-F4) and lowest (Control) number of different viruses. For each population, virgin adult females were isolated after emergence and were maintained in a plastic box separately from the males for 8 days. Water and food were provided ad libitum. On day 8 of adulthood, virgin females were collected and dissected with entomological tweezers under the binocular. First, the head and thorax of the flies were separated from the abdomen and conserved in RNA later (Sigma Aldrich R0901, St. Louis, MO, USA) as a somatic tissue sample. Then, the abdomen was opened vertically, visualizing the ovaries. Ovaries were carefully separated from other debris using sterile entomological pins and were conserved in RNA later (Sigma Aldrich R0901, St. Louis, MO, USA).

RNA was extracted from pools of 10 head/thorax and 20 ovaries using TriPure isolation reagent (cat. no. 11667157001; Roche, Mannheim, Germany). RNA integrity and quantity were assessed by 1% agarose gel electrophoresis and spectrophotometry. The viral prevalence and abundance in these samples were determined by RT-qPCR as described above.

Small RNA library construction and sequencing were carried out by Macrogen (Seoul, Korea). Libraries were prepared using the TruSeq Small RNA Library Prep Kit (Illumina, San Diego, CA, USA). Then, sequencing was performed in the HiSeqX platform (Illumina) generating paired-end reads of 150-nt long. Approximately 1 Gb of raw data were obtained from each of four the libraries analyzed (NCBI; SAMN21882268, SAMN21882269, SAMN21882270, and SAMN21882271).

### 2.7. Characterization of Viral sRNA Profiles

Adapters, empty sequences, low complexity sequences, and reads with more than 20% low-quality were removed from the raw RNA-Seq reads. Moreover, clean reads were filtered by length, maintaining only the sequences between 18 and 32 nt. Viral-derived sRNA sequences (vsRNAs) were obtained after mapping the clean reads against the genome sequences of the 13 RNA covert viruses described in medfly using Bowtie 2 v 2.3.5.1 [33]. The percentage of vsRNAs from each sRNA library mapping to each viral sequence was calculated after collapsing identical sequences. The mapping position of the sRNA reads along the viral sequences was visualized using an Integrative Genomics Viewer [36]. Length distribution, base composition, and strand distribution of the vsRNAs were analyzed to identify the predominant sRNA routes activated by the viral presence using a custom Python script described by Lewis et al., 2018 [37] (accessible on GitHub: https://github.com/SamuelHLewis/sRNAplot, accessed on 12 May 2021).

## 3. Results

### 3.1. RNA Virome Analysis Reveals the Presence of Thirteen RNA Viruses in Medfly

**Figure 1:**
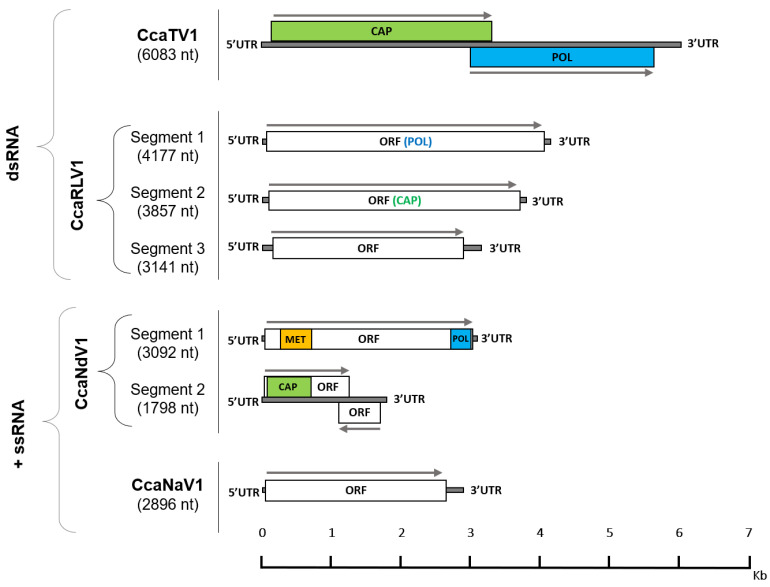
Putative genome organization of the four newly described viruses in medfly. Each genome is drawn proportionally to its length. White boxes represent open reading frames (ORFs) encoding viral proteins of an unknown function while colored regions represent ORFs encoding RNA-dependent RNA polymerase (POL, blue), capsid-protein (CAP, green), or methyltransferase (MET, yellow). The grey arrows displayed with the ORFs represent their orientation. Ceratitis capitata reo-like virus 1 (CcaRLV1) and Ceratitis capitata nodavirus 1 (CcaNdV1) harbor a segmented RNA genome, as shown for other viruses within their viral families.

In our aim to explore the RNA virome of medfly, we analyzed two different strains: control and W-1Kλ (Table S2). By identifying eight different viruses in their transcriptomes, four of them not previously described, we expanded the medfly virome to 13 viruses (Table 1). Sequence similarity with known viral sequences, genome structure, and phylogenetic analysis were used to confirm the viral nature of the new sequences and to classify them according to their family (Figure 1 and Figure S1 and Table S3).

Two new dsRNA viruses were identified and named as: Ceratitis capitata totivirus 1 (CcaTV1) and Ceratitis capitata reo-like virus 1 (CcaRLV1). CcaTV1 maintains the typical genome length (6132 bp) and organization of viruses in the *Totiviridae* family, with two ORFs encoding the capsid proteins and the RdRp, respectively (Figure 1). The phylogenetic analysis of the viral RdRp sequence revealed that CcaTV1 clustered together with other totiviruses infecting dipteran species such as Bactrocera dorsalis toti-like virus 1, Hubei toti-like virus 15, and Larkfield virus (Figure S1A). For CcaRLV1, three different segments of 4177-nt, 3857-nt, and 3141-nt were characterized. Each of them contained a single ORF encoding for the RdRp gene in segment 1, the putative major core protein gene in segment 2, and a peptide of unknown function in segment 3 (Figure 1). Presumably, additional CcaRLV1 viral sequences will be detected in the medfly, as members of the *Reoviridae* family generally contain 9 to 12 genome segments. The phylogenetic classification of CcaRLV1 segment 1 suggested a close relation of CcaRLV1 with the dipteran-infecting viruses Hubei Diptera virus 20, Bobbyc reo-like virus, and the rodent-infecting Bloomfield virus (Figure S1B).

In addition to the dsRNA viruses, two new positive ssRNA viruses were identified. A novel nodavirus (family *Nodaviridae*) and a new member of the *Narnaviridae* family were retrieved and annotated as Ceratitis capitata nodavirus 1 (CcaNdV1) and Ceratitis capitata narnavirus 1 (CcaNaV1), respectively. CcaNdV1 has a small segmented RNA genome with methyltransferase and RdRp encoded on the longer 3092-nt segment (Figure 1), as described for other members of the *Nodaviridae* family [38]. The phylogenetic analysis based on segment one showed a clustering of CcaNdV1 with other invertebrate-infecting alpha-nodaviruses (Figure S1C). The narnavirus CcaNaV1 has a 2896-nt genome, being the smallest of the RNA viruses discovered in medfly so far. It contains a single ORF in which no conserved domains have been identified (Figure 1). The phylogenetic analysis showed that CcaNaV1 clustered with Meagle narna-like virus and Soybean thrips narna-like virus 1, which infect insects of the orders Diptera and Thysanoptera, respectively (Figure S1D).

Other viruses discovered in our analyzed RNAseq data concern viruses recently described by other studies [7,8], although some of those viruses remained uncharacterized. Based on the phylogenetic analyses (Figure S1E), we propose including the previously described CcaTSA1 [8] within the *Virgaviridae* family under the name of Ceratitis capitata virga-like virus 1 (CcaViLV1). Similarly, we suggest the addition of CcaTSA2 and CcaTSA3 [7,8] into the unclassified negev-like viruses, and name them as Ceratitis capitata negev-like virus 1 (CcaNeLV1) and Ceratitis capitata negev-like virus 2 (CcaNeLV2), respectively (Table 1 and Figure S1E).

### 3.2. Prevalence and Viral Abundance Are Variable among Strains

We assessed the prevalence and relative abundance of the 13 RNA viruses in individuals and viral pre-purifications from two laboratory-reared medfly strains (“control” and “IVIA”), a mass-reared strain (“Vienna 8A”), a recently field-captured strain (“Wild-F4”) and in the *C. capitata* Cce-cc128 cell line. Viral pre-purifications were obtained from pools of 50 pupae to increase the concentration of viruses, as infections with certain RNA viruses could exhibit low viral titers.

**Figure 2:**
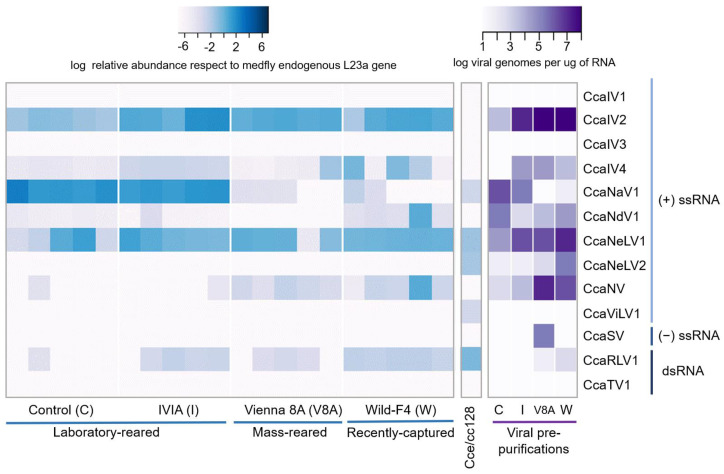
Relative virus abundance in different medfly strains and in a cell line. Heatmap of the relative abundance of the 13 medfly RNA viruses in five pupae (one pupa per column) from two laboratory-reared strains (control (C) and IVIA (I)), one mass-reared strain (Vienna 8A (V8A)), and one recently captured strain (Wild-F4 (W)), in the Cce/cc128 medfly cell line and in four viral pre-purifications obtained from each of the four previously mentioned strains. For the single pupae and the Cca/cc128 cell line, color plots represent values of viral relative abundance, calculated as viral RNA genomes relative to the expression of the mitochondrial L23a gene of medfly. For the viral pre-purifications, color plots represent viral abundance, calculated as viral genomes per µg of RNA using a standard curve with known viral concentrations.

Seven out of the 13 viruses were detected in medflies, with varying relative abundance per fly strain: CcaIV2, CcaIV4, CcaNV, CcaNaV1, CcaNdV1, CcaNeLV1, and CcaRLV1 (Figure 2). Two additional viruses, CcaNeLV2 and CcaSV, were detected only after viral pre-purification. Five RNA viruses were detected in the Cce/cc128 cell line, with similar levels of relative abundance: CcaNaV1, CcaNeLV1, CcaNeLV2, CcaRLV1, and CcaViLV1 (Figure 2). Three viruses were identified in all of the tested samples: CcaIV2, CcaIV4, and CcaNeLV1, with CcaIV2 and CcaNeLV1 showing higher values of relative abundance. The prevalence of CcaNaV1, CcaNdV1, CcaNV, and CcaRLV1 varied between strains and, in some cases, between flies within each strain (Figure 2)**.** Interestingly, the highest number of viral species, and the largest variation between individuals in viral titers and viral abundance were observed in the samples derived from the Wild-F4 strain (Figure 2).

**Figure 3:**
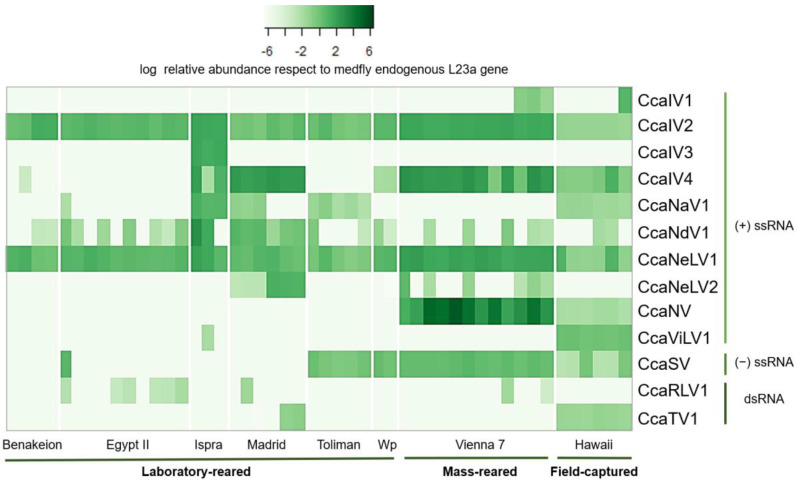
Viral relative abundance in sequence read archive (SRA) datasets. Heatmap of the relative abundance of the 13 medfly RNA viruses in 48 transcriptome datasets from eight different BioProjects available from NCBI. Color plot represents values of viral relative abundance calculated using the total viral counts and L23a mitochondrial gene counts, both determined by read mapping.

To complement the previous analysis, we additionally investigated 48 *C. capitata* RNAseq datasets available at NCBI for the presence of the 13 viruses described above. RNA sequencing data were selected from eight different BioProjects to represent up to eight diverse medfly strains or populations distributed worldwide: Benakeion (Greece), EgyptII (Egypt), Hawaiian (Hawai), Ispra (Italy), Madrid (Spain), Toliman (Guatemala), Vienna 7 (Austria), and Wp (Greece) (Table S2). Our results confirmed that each of the 13 RNA viruses were present in at least one of the examined datasets (Figure 3). CcaIV2 and CcaNeLV1 represent the core RNA virome of medfly, as they were retrieved in all of the analyzed datasets and in all of the analyzed fly strains (Figure 2 and Figure 3). On the contrary, CcaIV3 was exclusively detected in the Ispra strain (Italy). For the rest of the viruses, the prevalence and abundance were variable among the different fly strains (Figure 3). In addition, the dataset based on field-collected samples (Hawaii) harbored the largest number of different viruses, with 10 out of the 13 viruses being detected (Figure 3).

### 3.3. Small-Interfering RNA Pathway Targets Most of the Analyzed Viruses

**Figure 4:**
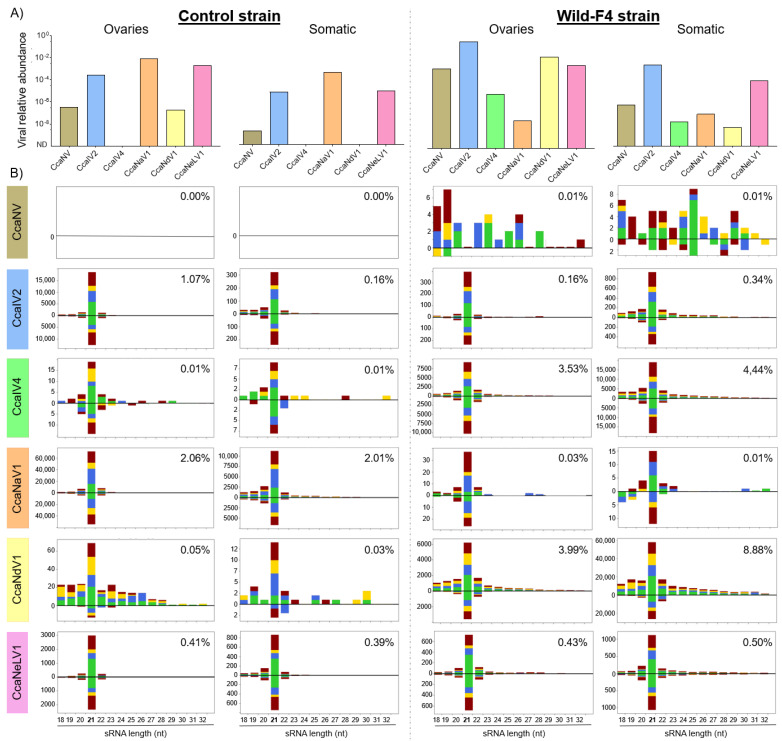
Viral relative abundance and small RNA profile of six positive ssRNA viruses in ovaries and somatic tissues of the control and Wild-F4 medfly strains. (**A**) The relative abundance of the six viruses was calculated using qPCR and referred to the expression of the endogenous L23a gene. (**B**) Size distribution of 18- to 32-nt small RNAs mapping to the viral genome of each of the six viruses. Positive-strand reads are displayed above the solid horizontal line, negative-strand reads are below. Percentages show the number of unique reads mapping to each of the viral genomes in comparison to the total unique sRNA reads account for each sRNA library. Colors indicate the first nucleotide of the sRNA read (red = U, green = A, blue = C, yellow = G).

Two medfly strains (control and Wild-F4) were selected for sRNA sequencing, and sRNA profiles were obtained for the ovaries (likely to play a role in vertical transmission) and the remaining somatic tissues. Viral relative abundance was evaluated in the four samples prior the sRNA library construction (Figure 4A). Five RNA viruses were detected in the two analyzed strains (CcaNV, CcaIV2, CcaNaV1, CcaNdV1, and CcaNeLV1), while one virus (CcaIV4) was retrieved only from the Wild-F4 strain (Figure 4A).

The sRNA profiles showed specific targeting of sRNAs to those viruses detected in the control and Wild-F4 strains. The abundance of the sRNA reads mapping to the different viral sequences ranged from 0% to 8.88% of the total sRNA reads (Figure 4B), and were correlated with the differences in viral abundance between samples (Figure 4A). The sRNA profiles exhibited a distinctive peak of 21-nt sRNAs for five positive ssRNA viruses: CcaIV2, CcaIV4, CcaNaV1, CcaNdV1, and CcaNeLV1. When present, these 21-nt sRNAs were mapped both to the positive and negative strands of the viral genomes, with the only exception being CcaNdV1 in the control strain, probably due to the low number of sRNA sequences. Mapping of sRNA reads to the viral genomes revealed a homogeneous distribution of the reads along the whole genomes (Figure S2). These results indicated that viral infection correlates with the presence of viral-derived siRNAs in both control and Wild-F4 strains, regardless of the tissue analyzed. Moreover, the siRNA profile is maintained for all these viruses independently from their viral relative abundance and the percentage of sRNA reads mapping to the viral genomes. In contrast, although CcaNV was found in both strains and both tissues (Figure 4A), and the characteristic siRNA profile was absent in the control and Wild-F4 strains. Few sRNA sequences were mapped to the CcaNV genome in the Wild-F4 strain, but with a random length distribution, probably produced by unspecific degradation of viral RNA (Figure 4B). Altogether, these results suggest that, differently from other positive ssRNA viruses, CcaNV does not trigger the RNAi response in medfly (Figure 4B and Figure S2).

## 4. Discussion

By means of transcriptome mining and viral genome annotation, we have obtained and characterized the largest RNA virome of medfly described to date [7,8,10,14]. This work expands the medfly RNA virome to 13 different viruses by the addition of four new viruses: two positive ssRNA viruses (CcaNaV1 and CcaNdV1) and the first two dsRNA viruses described for medfly (CcaRLV1 and CcaTV1). Based on the large number of ISVs recently discovered in other arthropod species boosted by the use of high throughput sequencing methods [3,7,8,39], the existence of additional RNA viruses infecting medflies cannot be discarded. In this context, the higher viral diversity in field-captured samples observed in our study suggests that screening of additional field samples would likely expand the medfly virome. Nevertheless, further research will be needed to confirm this hypothesis and to elucidate whether the transition from field to laboratory rearing conditions can trigger the clearance of some RNA viruses. In fact, previous reports in tsetse flies [40] and of the tephritid fruit flies *Bactrocera tryoni* and *Bactrocera cacuminata* [7] strikingly contrast with this hypothesis, revealing a higher viral diversity and prevalence in laboratory-reared samples.

The viral prevalence and abundance of the 13 identified RNA viruses corroborated the presence of multiple and simultaneous viral infections in medflies [10] and revealed that CcaIV2 and CcaNeLV1 were ubiquitous in the 12 analyzed medfly strains, and were hence defined as the core RNA virome of medfly (Figure 2 and Figure 3). This could be the result of a long-term co-evolution of these viruses with the host. Alternatively, it is tempting to hypothesize that CcaIV2 and CcaNeLV1 could provide an evolutionary advantage for the flies. Examples of mutualistic relations between viruses and their insect hosts have been recently described in other multitrophic systems [41,42]. For instance, partiti-like viruses (dsRNA) exert a positive effect on *S. exempta* fitness by increasing its resistance against baculovirus infection [2]. In another example, Acyrthosiphon pisum virus (positive ssRNA) infection in aphids enhanced their adaptation to unsuitable host plants by regulating the phytohormone defense response elicited by the colonized plant [43].

While the lack of medfly strains free from specific viruses limits the study of functional associations, the characterization of the immune responses elicited in the host against viral infections may contribute to elucidating the outcome of the virus–host interactions. According to our results, the siRNA pathway is active against RNA viruses in medflies, as was previously observed in *Ae. vexans arabiensis* mosquitoes and the oriental fruit fly *B. dorsalis* [6,20]. Except for CcaNV, viral-derived siRNAs of 21-nt were the most abundant sRNAs in both the ovaries and somatic tissue of Wild-F4 and control medfly strains. In general terms, our data showed that a higher vsiRNA production correlates with a higher viral abundance, while no vsiRNAs were found for the viruses absent in the samples (Figure 4). Differently from the rest of the detected viruses, the presence of CcaNV in the Wild-F4 and control strains did not correlate with the production of vsiRNAs. Viral suppression of the antiviral RNAi pathway has been previously demonstrated for a variety of plant and insect hosts. The VP1 protein is one of the conserved proteins among the different Nora virus members, including CcaNV. VP1 from Drosophila melanogaster Nora virus suppresses the host RNAi by inhibiting AGO2 slicer activity [44]. Interestingly, so far, CcaNV is the only virus likely associated with a decrease in medfly fitness by reducing the adult lifespan [10]. The production of vsiRNAs for other medfly RNA viruses suggests that the mechanism used by CcaNV to mediate RNAi suppression is virus-specific and does not interfere with the host response to the other viral infections. However, we cannot discard the possibility of CcaNV being physically isolated from the other viruses by infecting different tissues or cells, although all viruses were detected in the ovaries. In this context, further analysis of the spatiotemporal expression of RNA viruses will be needed to clarify whether multiple RNA viruses can simultaneously infect the same cells and tissues of the fly.

In summary, we have expanded the RNA virome of medfly and revealed the ubiquitous distribution of two medfly viruses (CcaIV2 and CcaNeLV1) in different medflies worldwide. Unraveling the impact of these viruses on medfly fitness, including their influence on medfly interactions with third trophic level insects, will allow us to determine the impact of this viruses on shaping medfly ecology and contribute to decipher their potential pathogenic risk for medfly mass-rearing and their impact on sterile males released into the field for SIT programs.

## Figures and Tables

**Table 1:** List of the 13 RNA viruses described for medfly.

Virus Type	Virus Family	Virus Full Name	Virus Abbreviation	Genome Length (bp)	Genbank Accession Numbers
positive ssRNA	*Iflaviridae*	Ceratitis capitata iflavirus 1	CcaIV1	10,502	GAMC01001920.1
		Ceratitis capitata iflavirus 2	CcaIV2	10,332	OL957305
		Ceratitis capitata iflavirus 3	CcaIV3	7370	HG994137
		Ceratitis capitata iflavirus 4	CcaIV4	9023	HG994138
	*Narnaviridae*	Ceratitis capitata narnavirus 1	CcaNaV1	2896	OL957306
	*Nodaviridae*	Ceratitis capitata nodavirus 1, segment 1	CcaNdV1_s1	3092	OL957308
		Ceratitis capitata nodavirus 1, segment 2	CcaNdV1_s2	1798	OL957309
	*Negeviridae*	Ceratitis capitata negev-like virus 1	CcaNeLV1	10,258	HG994139
		Ceratitis capitata negev-like virus 2	CcaNeLV2	10,506	OL957307
	*Unclassified Riboviria*	Ceratitis capitata nora virus	CcaNV	12,014	GAMC01015827.1
	*Virgaviridae*	Ceratits capitata virga-like virus 1	CcaViLV1	9925	GAMC01017950.1
negative ssRNA	*Rhabdoviridae*	Ceratitis capitata sigmavirus	CcaSV	12,583	KR822825.1
dsRNA	*Reoviridae*	Ceratitis capitata reo-like virus 1, segment 1	CcaRLV1_s1	4177	OL957310
		Ceratitis capitata reo-like virus 1, segment 2	CcaRLV1_s2	3857	OL957311
		Ceratitis capitata reo-like virus 1, segment 3	CcaRLV1_s3	3141	OL957312
	*Totiviridae*	Ceratitis capitata totivirus 1	CcaTV1	6132	OL957313

## Data Availability

The data presented in this study are openly available in the National Center of Biotechnology Information (NCBI). Accession numbers for raw transcriptomic data are contained in Table S2. Accession numbers for the nucleotide sequence of the new viruses described in medfly are included in Table 1. Accession numbers for the protein sequences included in the phylogenetic analysis can be found in Table S3.

## Supplementary Materials

The following supporting information can be downloaded at: https://www.mdpi.com/article/10.3390/v14030623/s1. Figure S1: Phylogenetic classification of the newly described medfly covert RNA viruses. Figure S2: Mapping of the sRNA reads to the viral genomes. Table S1: List of the 13 RNA viruses described in the medfly and the primers designed to amplify them through RT-qPCR. Table S2: List of 48 medfly sequence read archives (SRAs) queried for viral presence and relative abundance using in silico methods. Table S3: List of viral proteins included in the phylogenetic analysis.
