# Supplementary material for: Expanding the Medfly Virome: Viral Diversity, Prevalence, and sRNA Profiling in Mass-Reared and Field-Derived Medflies
Source: Viruses. 2022 Mar 17;14(3):623. doi: 10.3390/v14030623 (PMC8955247; doi:10.3390/v14030623)
Supplement: Supplementary file 1 [file viruses-14-00623-s001.zip › viruses-1554753-supplementary.pdf]

Supplementary Materials:

Supplementary Figure S1:

A) Ceratitis capitata totivirus 1

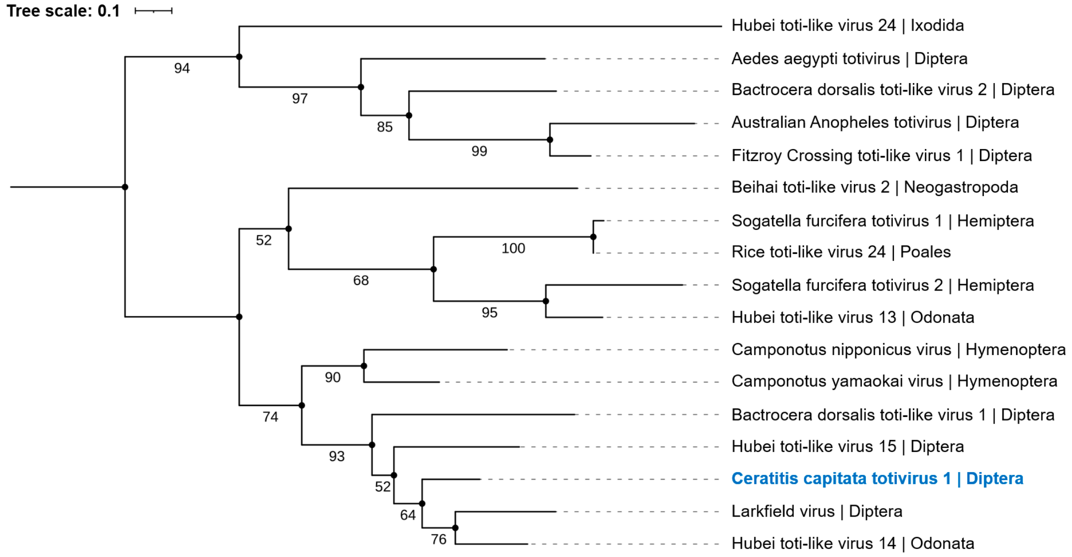

B) Ceratitis capitata reo-like virus 1

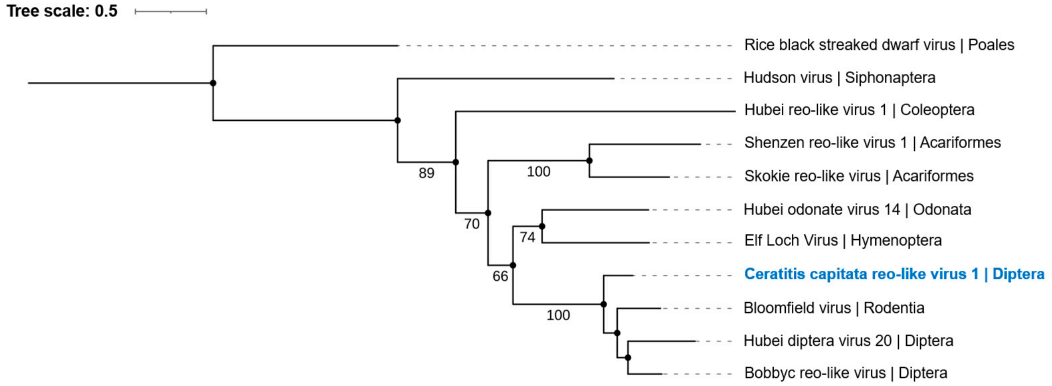

C) Ceratitis capitata nodavirus 1

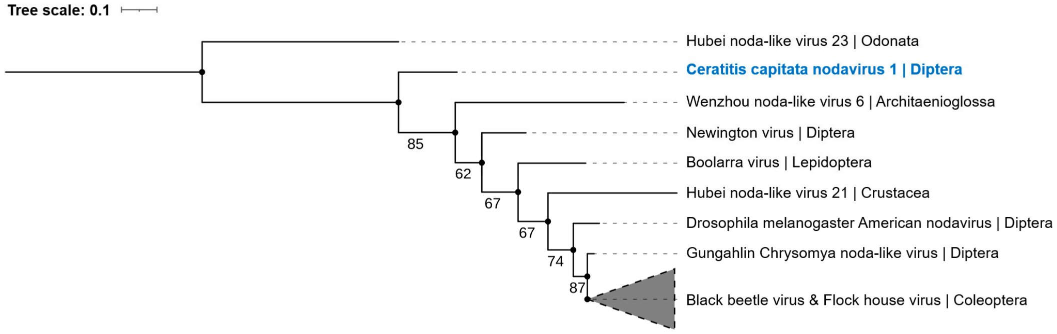

D) Ceratitis capitata narnavirus 1:

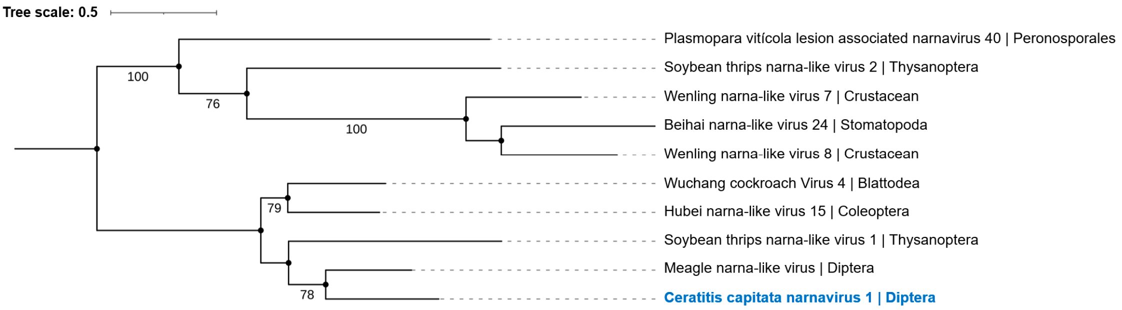

E) Ceratitis capitata virga-like virus 1; Ceratitis capitata negev-like virus 1, and Ceratitis capitata negev-like virus 2:

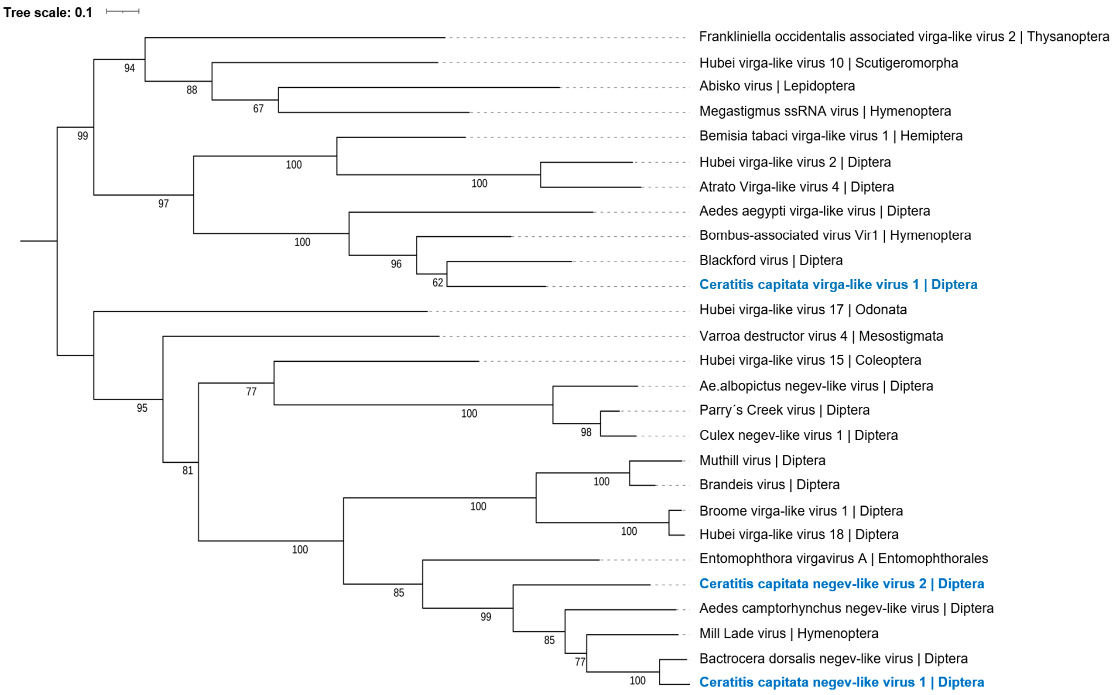

Figure S1 **Phylogenetic classification of the newly described medfly covert RNA viruses.** Sequences were analyzed at the amino acid level and the closest viral relatives to the medfly viruses were detected using blastp (Table S3). Phylogenetic inferences were calculated using IQtree2 with default settings and 1000 ultrafast bootstrap. RNA viruses infecting medfly are shown in blue in the figures.

Supplementary Figure S2:

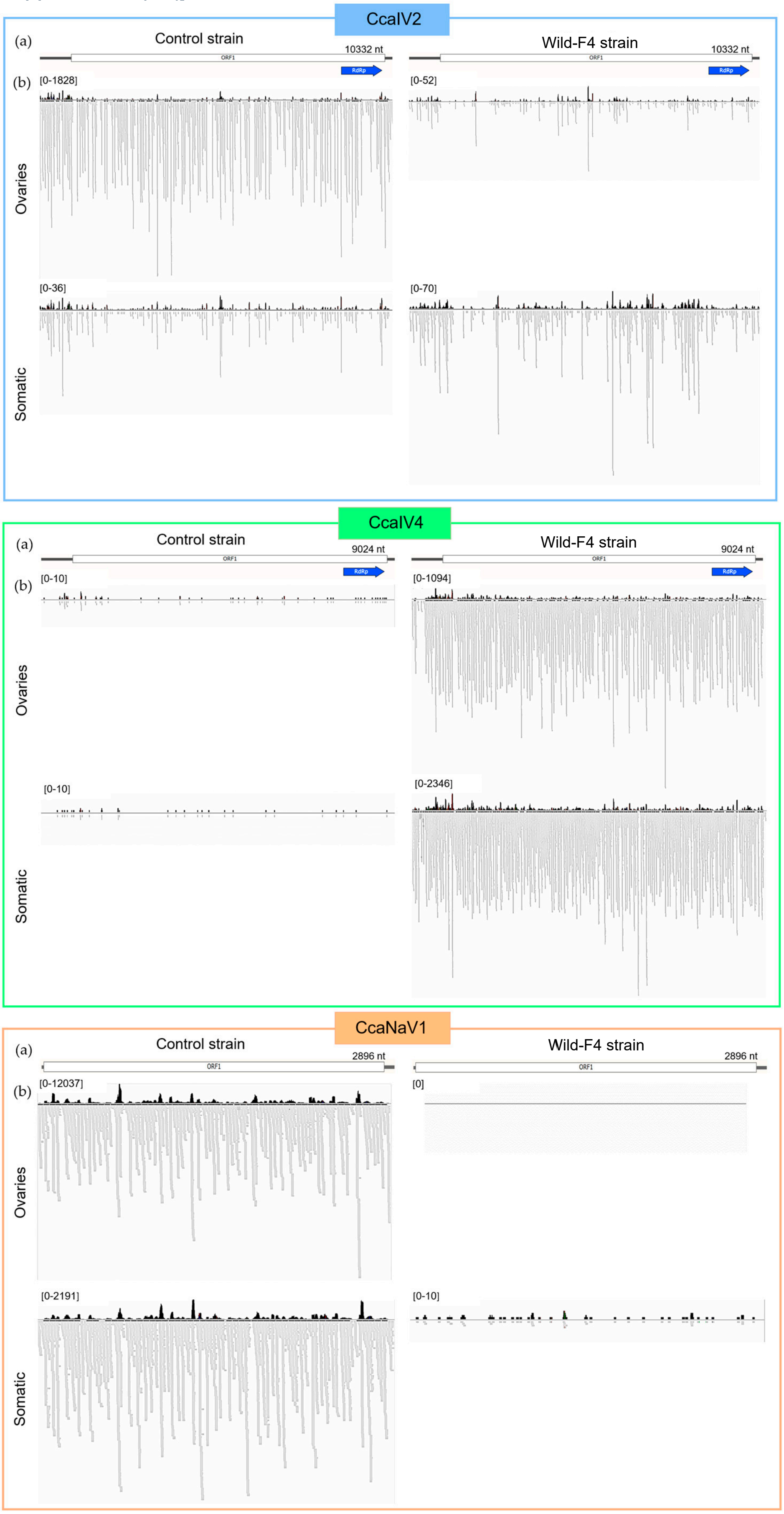

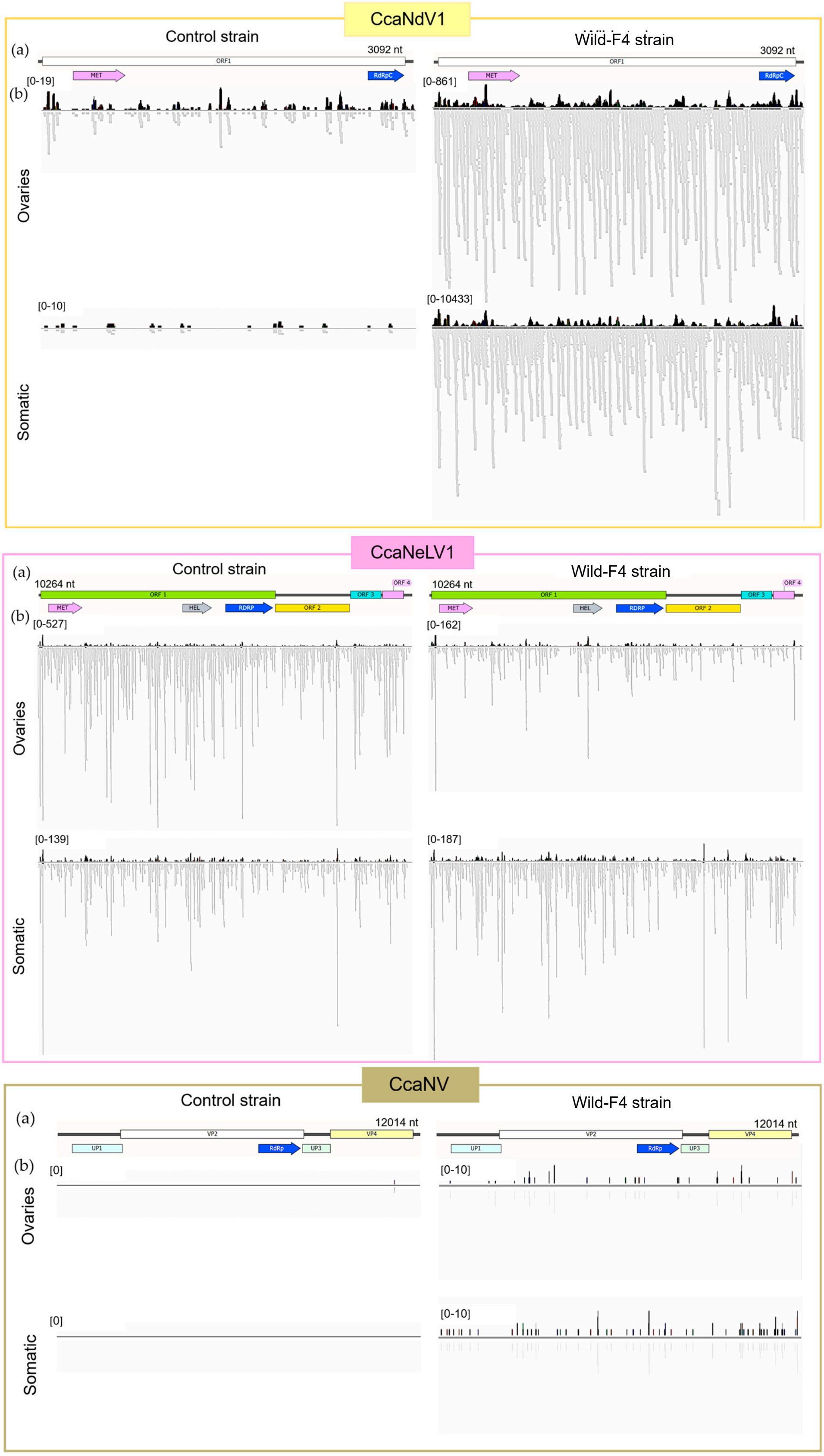

Supplementary Figure S2 **Mapping of the sRNA reads to the viral genomes.** (a) Viral sequence including open reading frames (square) and predicted domains (arrows). (b) Distribution of the small RNAs along the sequence of the six viruses detected in ovaries and somatic tissues of Control and Wild strains of medfly.

Supplementary Table S1 **List of the 13 RNA viruses described in the medfly and the primers designed to amplify them through RT-qPCR**.

Virus	Genome length (bp)	NCBI accession number	Forward primer	Reverse primer	Estimated efficiency (%)
**CcaIV1**	10502	GAMC01001920.1	TATGTGTTGTCCACCCCAGC	TGTGCGCATGGTGATTCTAAC	-
**CcaIV2**	10332	OL957305	CCAAGATAAGAATAGGCTAATGCGT	AACATTCGCATTTACCATTAACACAG	110.731
**CcaIV3**	7370	HG994137	GATGTTGGTGAGTTCCTC	CTATTGCCGTTAGTGAGAC	-
**CcaIV4**	9023	HG994138	CGTTGGTTGTTTGATTTGGAAG	GTGAAGATTGTTGGCGGAAC	131.196
**CcaNaV1**	2896	OL957306	GCTCCATCTTGGGTTCATTTG	CTGCCGTCTCCATCTTTCC	100.707
**CcaNdV1**	3092	OL957308	CTAATCGGTATGGGACAC	GACAAACTGGAGACACAAC	114.666
	1798	OL957309	-	-	-
**CcaNeLV1**	10258	HG994139	GCGGGTAGAAGAGTATTG	GACATTCGCAGATAACAG	118.508
**CcaNeLV2**	10506	OL957307	CCCATCTAACCCTACTCC	GCGTTCTTCTTGTCATTC	117.12
**CcaNV**	12014	GAMC01015827.1	GCTCAAGCAAAAGCAGGCAG	AGCATTTCAGTCCGCTACGA	110.981
**CcaViLV1**	9925	GAMC01017950.1	ATGATACGGTTGACTTTCAG	GAGGTGGTGACGAGTAAG	109.206
**CcaSV**	12583	KR822825.1	TACAAACAAACACGAAGC	CACACCCATAACCTACAG	-
**CcaRLV1**	4177	OL957310	GACTAACAGGTCTCAAGG	CGCTACTCTAACTTGACC	94.234
	3857	OL957311	-	-	-
	3141	OL957312	-	-	-
**CcaTV1**	6132	OL957313	CTCAAGGAAGAGACACAG	GAGATATGTCTGGCGTAG	-
**L23a**	991	XM004518966	GCCGAGAAATCCGCCAAATC	CTTTGCTGCAGCGGGTTTAG	104.17

Supplementary Table S2 **List of 48 medfly sequence read archives (SRAs) queried for viral presence and relative abundance using *in silico* methods**.

NCBI accession number	Sequencing method	*C. capitata* origin	Library details		SRA accession number
SRP133427	Illumina HiSeq 2000	Benakeion	10 adults, whole body	10 adult M	SRR6780762
				10 adult F	SRR6780764
ERP119522	Illumina NovaSeq 6000; pair end		Pool of 3 larvae	Immobilized larvae	ERR4026379
				Jumping larvae	ERR4026376
SRP073787	Illumina HiSeq 2000, single	Egypt II	Gastrointestinal tract	Virgin female	SRR3436822
			Reproductive tract	Mated male	SRR3436821
				Mated male	SRR3436820
				Mated female	SRR3436819
				Mated female	SRR3436818
				Virgin male	SRR3436817
				Virgin male	SRR3436816
				Virgin female	SRR3436815
				Virgin female	SRR3436814
			Gastrointestinal tract	Virgin female	SRR3436813
SRP021914	Illumina Genome Analyzer Iix; pair end	Ispra	Adult	Male	SRR836190
				Female	SRR836189
			Embryo		SRR836188
ERP124511	Illumina HiSeq 2000 paired end sequencing	Madrid	Whole adult, Control strain	Replicate 1	ERR4690327
				Replicate 2	ERR4690326
				Replicate 3	ERR4690325
SRP343008			Whole adult, W-1Kλ strain	Replicate 1	SRR16562767
				Replicate 2	SRR16562768
				Replicate 3	SRR16562769
SRP075464	Illumina Genome Analyzer II	Toliman	Female ovary		SRR3554596
			Elongated spermatids		SRR3554595
			Round spermatids		SRR3554594
			Late spermatocytes		SRR3554593
			Early spermatocytes		SRR3554592

ERP119522	Illumina NovaSeq 6000; pair end	Wp	Pool of 3 larvae	Immobilized larvae	ERR4026385
				Jumping larvae	ERR4026382
SRP026213	Illumina HiSeq 2000	**Vienna 7**	Non-irradiated	Pupae	SRR915844
					SRR915840
					SRR915839
				Adult	SRR915838
					SRR915837
					SRR915836
			Irradiated	Pupae	SRR915835
					SRR915834
					SRR915833
				Adult	SRR915832
					SRR915831
					SRR915830
SRP026213	Illumina HiSeq 2000	**Hawaiian**	Whole body	Wild pupae	SRR915852
					SRR915851
					SRR915850
				Wild adults	SRR915848
					SRR915846
					SRR915845

Supplementary Table S3 **List of viral proteins included in the phylogenetic analysis.**

Viral name	Protein accession number	Viral name	Protein accession number
**(A)** ***Totiviridae***		**(D)** ***Narnaviridae***	
Aedes aegypti totivirus	BBM96510.1	Beihai narna-like virus 24	YP_009333245.1
Australian Anopheles totivirus	ASU43981.1	Ceratitis capitata narna-like virus 1	*OL957306
Bactrocera dorsalis toti-like virus 1	QMU95572.1	Hubei narna-like virus 15	YP_009337783.1
Bactrocera dorsalis toti-like virus 2	QMU95575.1	Meagle narna-like virus	QIJ70070.1
Beihai toti-like virus 2	APG75984.1	Plasmopara viticola lesion associated narnavirus 40	QIR30319.1
Camponotus nipponicus virus	YP_009230208.1	Soybean thrips narna-like virus 1	QQP18719.1
Camponotus yamaokai virus	YP_009143313.1	Soybean thrips narna-like virus 2	QQP18720.1
Ceratitis capitata totivirus 1	*OL957313	Wenling narna-like virus 7	YP_009337166.1
Fitzroy Crossing toti-like virus 1	QLJ83495.1	Wenling narna-like virus 8	APG77263.1
Hubei toti-like virus 13	YP_009336780.1	Wuchang cockroach Virus 4	YP_009342297.1
Hubei toti-like virus 14	APG76023.1	***(E) Negeviridae***	
Hubei toti-like virus 15	YP_009336859.1	Abisko virus	YP_009408586.1
Hubei toti-like virus 24	YP_009336908.1	Ades albopictus negev-like virus	QGN03453.1
Larkfield virus	AWA82248.1	Aedes aegypti virga-like virus	BBN20999.1
Rice Toti-like virus	QKN84381.1	Aedes camptorhynchus negev-like virus	YP_009388601.1
Sogatella furcifera totivirus 1	YP_009552726.1	Atrato Virga-like virus 4	QHA33744.1
Sogatella furcifera totivirus 2	YP_009553182.1	Bactrocera dorsalis negev-lik virus	QMU95554.1
**(B)** ***Reoviridae***		Bemisia tabaci virga-like virus 1	QNJ34552.1
Bloomfield virus	AWB14602.1	Blackford virus	KU754514.2
Bobbyc reo-like virus	QIJ70114.1	Bombus-associated virus Vir1	QAY29261.1
Ceratitis capitata reo-like virus 1	*OL957310	Brandeis virus	AVZ66283.1
Elf Loch virus	QAY29252.1	Broome virga-like virus 1	QLJ83498.1
Hubei diptera virus 20	APG79173.1	Ceratitis capitata Negev-like virus 1	*HG994139
Hubei odonate virus 14	APG79163.1	Ceratitis capitata Negev-like virus 2	*OL957307
Hubei reo-like virus 1	APG79117.1	Ceratitis capitata Paretaria Mottle-like virus	*GAMC01017950.1
Hudson virus	QED21522.1	Culex negev-like virus 1	YP_009388585.1
Rice black streaked dwarf virus	QFO48930.1	Entomophthora virgavirus A	QED42956.1
Shenzhen reo-like virus 1	QPN36950.1	Frankliniella occidentalis associated virga-like virus 2	QNM37806.1
Skokie reo-like virus	QPG92977.1	Hubei virga-like virus 10	APG77802.1
(C) Nodaviridae		Hubei virga-like virus 15	YP_009337693.1
Black beetle virus	YP_053043.1	Hubei virga-like virus 17	YP_009337715.1
Boolarra virus	NP_689439.1	Hubei virga-like virus 18	YP_009333286.1
Ceratitis capitata nodavirus 1	*OL957308	Hubei virga-like virus 2	YP_009337412.1
Drosophila melanogaster American nodavirus (ANV) SW-2009a	ACU32794.1	Megastigmus ssRNA virus	QDZ71189.1
Flock House virus	NP_689444.1	Mill Lade virus	QAY29259.1
Gungahlin Chrysomya noda-like virus	QIJ70031.1	Muthill virus	AMO03223.1
Hubei noda-like virus 21	APG76486.1	Parry's Creek negev-like virus 1	QLJ83503.1
Hubei noda-like virus 23	APG76332.1	Varroa destructor virus 4	QGA69815.1
Newington virus	AMO03244.1		
Wenzhou noda-like virus 6	APG76600.1		

*Nucleotide accession numbers were provided when protein accession numbers were not available. Nucleotide sequences were translated into protein using the standard code.

Supplementary Table S4. **Total number of sRNA reads mapping to each RNA virus and the percentage they represent respect to the total number of reads**.

	C. capitata Control strain				*C. capitata* Wild-F4 strain			
	**Somatic tissue** (322209 sRNA reads)		**Ovaries** (665240 sRNA reads)		**Somatic tissue** (454562 sRNA reads)		**Ovaries** (286966 sRNA reads)	
	Aligned reads	%	Aligned reads	%	Aligned reads	%	Aligned reads	%
CcaIV1	5	0	41	0,010	11	0	5	0
CcaIV2	511	0,160	7118	1,070	1563	0,34	465	0,16
CcaIV3	1	0	37	0,010	3	0,	1	0,00
CcaIV4	29	0,010	57	0,010	20182	4,44	10116	3,53
CcaNaV1	6479	2,010	13672	2,060	46	0,01	78	0,03
CcaNdV1	91	0,030	313	0,050	40351	8,88	11451	3,99
CcaNeLV1	1269	0,390	2738	0,410	2261	0,50	1226	0,43
CcaNeLV2	2	0	2	0	1	0	0	0
CcaNV	0	0	2	0	67	0,01	28	0,01
CcaRLV1	1	0	0	0	92	0,02	0	0

Four datasets were analyzed, representing somatic tissue and ovaries samples from "Control" and "Wild-F4" medfly strains. The distribution of these reads along the genomes of CcaIV2, CcaIV4, CcaNaV1, CcaNdV1, CcaNeLV1, and CcaNV is displayed in Figure S2. No sRNA reads mapping to CcaSV, CcaTV1, and CcaViLV1 were found.
